# Data Sharing in a Decentralized Public Health System: Lessons From COVID-19 Syndromic Surveillance

**DOI:** 10.2196/52587

**Published:** 2024-03-28

**Authors:** Ryan C Rigby, Alva O Ferdinand, Hye-Chung Kum, Cason Schmit

**Affiliations:** 1 Program in Health, Law, and Policy Department of Health Policy and Management Texas A&M University School of Public Health College Station, TX United States; 2 Department of Health Policy and Management Texas A&M University School of Public Health College Station, TX United States; 3 Population Informatics Lab Department of Health Policy and Management Texas A&M University School of Public Health College Station, TX United States

**Keywords:** syndromic surveillance, federalism, COVID-19, public health, SARS-CoV-2, COVID-19 pandemic, United States, decentralized, data sharing, digital health, ethical guidelines, risk score, technology, innovation, information system, collaborative framework, infodemiology, digital technology, health information, health data, health policy, surveillance

## Abstract

The COVID-19 pandemic revealed that data sharing challenges persist across public health information systems. We examine the specific challenges in sharing syndromic surveillance data between state, local, and federal partners. These challenges are complicated by US federalism, which decentralizes public health response and creates friction between different government units. The current policies restrict federal access to state and local syndromic surveillance data without each jurisdiction’s consent. These policies frustrate legitimate federal governmental interests and are contrary to ethical guidelines for public health data sharing. Nevertheless, state and local public health agencies must continue to play a central role as there are important risks in interpreting syndromic surveillance data without understanding local contexts. Policies establishing a collaborative framework will be needed to support data sharing between federal, state, and local partners. A collaborative framework would be enhanced by a governance group with robust state and local involvement and policy guardrails to ensure the use of data is appropriate. These policy and relational challenges must be addressed to actualize a truly national public health information system.

## Introduction

Dr Rochelle Walensky, director of the Centers for Disease Control and Prevention (CDC), has admitted that the national response to the COVID-19 pandemic was deficient in many respects, citing the use and dissemination of data as a principal problem [1]. Indeed, the US public often relied on private data sources, such as the Johns Hopkins Coronavirus Resource Center, as the “go-to data source” for COVID-19 data rather than official government sources [2]. Many of the challenges concerning the use and dissemination of data are not due to the need to collect missing data, nor due to the technical limitations in sharing the needed data. Rather, many of these challenges are due to policy barriers that impede the needed data flows between public health entities, including local, state, and federal partners [3].

While data sharing challenges exist across the public health surveillance and dissemination systems, here we examine the specific challenges faced when sharing data between state, local, and federal syndromic surveillance partners. These challenges have been explored from state perspectives, but we examine these challenges through a national lens and how federalism issues affect federal, state, and local partners and shape the data use agreements (DUAs) that govern the data sharing relationships [4,5].

## US Syndromic Surveillance and Federalism

Syndromic surveillance refers to the process of sharing electronic data with health departments—often in near real time—to understand existing and emerging public health issues [6]. This process can include diverse data sources, including emergency department electronic health records (EHRs), environmental data, vital statistics, and laboratory data. The rapid nature of syndromic surveillance permits real-time situational awareness of emergent public health issues [6].

The United States began its investment in syndromic surveillance after the terrorist attacks of September 11, 2001, and a rash of anthrax-laced letters [7,8]. In 2009, syndromic surveillance was included as part of the US $20 billion EHRs incentive program created by the Health Information Technology of Economic and Clinical Health (HITECH) Act [9]. The Act incentivized hospitals to adopt, and meaningfully use certified EHR systems. The Act also accelerated syndromic surveillance nationally by incentivizing hospitals to report specific public health measures, including syndromic surveillance, to their local public health authorities [10]. Over this time, the national syndromic surveillance system, which was first developed by CDC, evolved from BioSense to BioSense 2.0 to the National Syndromic Surveillance System (NSSP), with the overall purpose of establishing a nationwide surveillance system to detect and assess potential health outbreaks throughout the United States [7]. Each of these evolutions has been impelled by technical, policy, and relational challenges between federal and state or local syndromic surveillance partners, including CDC, Council of State and Territorial Epidemiologists (CSTE), Association of State and Territorial Health Officials, and National Association of County and City Health Officials.

The community of practice, one of the defining aspects of the NSSP, aims to improve syndromic surveillance nationally through collaboration and knowledge sharing between federal, state, and local partners [11]. The community of practice offers a platform for public health professionals to collectively identify and use the optimal approach in syndromic surveillance. Through professional engagements, members collaborate to exchange knowledge, improve understanding, cultivate expertise, and address issues, to advance the practice of syndromic surveillance [11].

Currently, the NSSP stores all participating state and local syndromic surveillance data in a single repository maintained by the CDC [6]. While some federal employees have access to all syndromic surveillance to provide technical system support for state and local jurisdictions (eg, quality assurance, troubleshooting, and developing analytical tools), these data are not shared by default for federal public health uses [5]. The DUAs between state or local governments and the CDC prohibit any federal access to state and local data for public health purposes without the express consent of the jurisdictions [4,5]. Specifically, the default federal access to syndromic surveillance data for public health purposes is limited to the US Department of Health and Human Services (HHS) region level. For example, while federal NSSP personnel might observe an incidence increase within HHS Region 10 comprising Alaska, Idaho, Oregon, and Washington, they could not distinguish between (1) an isolated event in Washington; (2) unrelated, but similar, events in Washington and Alaska; or (3) related events in Washington and Oregon [5,12]. At the regional level, the federal government lacks awareness of interstate public health events. These policy data sharing limitations effectively hamstrung the federal COVID-19 response by not allowing access to vital data that could have been used to give a broader view to federal agencies in the context of a novel, rapidly moving and evolving virus.

The COVID-19 data sharing challenges cannot be understood without fully appreciating how US federalism shapes and affects the US public health system. In the United States, federalism consists of the sharing of authority between the national (federal) government and the state governments. At the onset, it is important to recognize that there is no express public health power that is explicitly outlined in the US Constitution. This means that federal public health actions must derive from one of the US Constitution’s enumerated powers. Typically, most federal public health actions are derived from the powers to tax and spend for the “general welfare” and the interstate commerce clause (ie, regulation of industries and activities that affect interstate and international commerce, including health).

The police power—the states’ power to regulate in the interest of public health, safety, and community values—is the fundamental governmental power that authorizes nearly all traditional public health actions [13]. Under the Constitution’s 10th Amendment, these powers are reserved for the states. This means that the states have the authority to set quarantines, restrict businesses, mandate isolation for those infected with a communicable disease, and impose primary public health responsibility [14].

In comparison, the federal government’s role is much more limited to providing support to state and local governments (ie, taxing and spending for the general welfare) or addressing those issues that have interstate impacts, like vaccine approvals (ie, interstate commerce). Consequently, state and local governments can be thought to have the primary public health responsibility within their jurisdictions while the federal government has less direct public health responsibilities and interests (ie, supporting state and local governments and interstate commerce).

These federalism nuances and the consequent relationships between local, state, and federal partners came under strain during the COVID-19 response. Specifically, the DUAs between the CDC and the states effectively impeded the response to the COVID-19 pandemic by restricting federal access to HHS region level syndromic surveillance data [15]. In early 2020, the White House COVID-19 Task Force obtained access to all COVID-19 NSSP data in an apparent exercise of emergency authority [4,5]. Although the federal government was able to bypass these policy barriers, the decision left some state and local epidemiologists feeling that the agreed-upon DUAs were “thrown out the window” [5].

While examining the US legal framework for data sharing within US federalism, Fahey [16] writes that data sharing is a rapidly expanding intergovernmental marketplace in many areas of government, including public health. She argues that in the absence of federal legislation, documents such as DUAs are the strongest legal policies [16]. The consequences and the reality of DUAs being discarded in the event of a national emergency are something that will only become more complex as data collection and data sharing increases. The power that federalism gives the states in these circumstances may become harder to define if clear policy regarding data is not codified through binding statutes.

## Improving the National Public Health Data System for Future Epidemics

In the following sections, we derive important lessons learned about public health data sharing within the US public health system, derived from the findings from a 2021 study by CSTE and the NSSP [4,5]. The study involved several work group calls with 20-30 state and local epidemiologists in leadership or decision-making roles and 8 key informant interviews. In addition, the study cited 8 randomly selected state and local epidemiologists from the list of NSSP site administrators to provide feedback on specific policy options [5].

## Align US Public Health Data Sharing Policies With Ethical Guidelines

An increasing number of public health ethicists assert that there are ethical obligations to share public health surveillance data in certain circumstances [17-19]. For example, the World Health Organization’s (WHO) ethical guidelines expressly state [18]:

[w]ith appropriate safeguards and justification, those responsible for public health surveillance have an obligation to share data with other national and international public health agencies.

Surveillance data can be legitimately disseminated to enable public health response, improve the efficiency and effectiveness of public health activities, and inform resource allocation and other support [20].

By limiting federal access to state and local NSSP data by default, the DUAs between the CDC and the states create significant impediments to legitimate public health functions under the enumerated powers to regulate interstate commerce and to spend for the general welfare [13]. In the context of a rapidly spreading public health threat such as the COVID-19 pandemic, the burden on the federal government to seek consent from all affected jurisdictions, each of which are also burdened by local public health actions, is antithetical to the calls for increased public health data sharing from ethicists. These DUAs should be revised to eliminate data sharing barriers when there is a documented and communicated public health need and there are appropriate policy guardrails to ensure only appropriate public health uses them. Importantly, proper guardrails can also eliminate other data sharing barriers [21].

## Using Granular Data to Respond to Interstate Threats and Allocate Federal Resources Equitably

The NSSP DUAs that restrict federal access to state and local syndromic surveillance data impede several legitimate federal interests and national public health objectives. The DUAs permit routine federal access only to HHS region level aggregations [5]. In order to have a national view of emerging public health threats, access to data at the state level or at a more granular level is required.

A national view of public health threats is essential for the federal government to fulfill its legitimate governmental role in national public health responses [5]. The federal government plays a critical role in providing support and allocating resources to state and local jurisdictions. However, allocating these resources equitably in response to national public health events requires broad situational awareness of the burdens faced by all jurisdictions. This type of assessment cannot occur when the federal government lacks the data at the required level of granularity. This proved to be one of the major challenges during the COVID-19 pandemic [22].

Moreover, several state and local epidemiologists expressed openness to increased federal access to state and local NSSP data to enhance their public health actions. For example, a commonly expressed benefit among key informants was having extra “eyes” on data to provide greater detection capacity. One epidemiologist stated, “I do think there’s also a lot of opportunity right now, [but] there’s not enough capacity...to look at local data” [5]. Other articulated anticipated benefits were more coordination between agencies and the generation of regular reports or visualizations based on agreed-upon queries of state and local NSSP data [5]. Additionally, enabling greater access to the CDC could permit the creation of national training programs to train and support a growing number of state and local epidemiologists in syndromic surveillance methods and techniques.

## State and Local Agencies Remain Essential Partners in Public Health Surveillance Activities

Regardless of legitimate federal interests, state and local governments still need to play a central role in syndromic surveillance for several reasons. First and foremost, state and local governments retain the primary public health responsibility for the communities within their jurisdictions. As a result, the duty to use syndromic surveillance data to improve population health in their jurisdictions rests squarely with state and local jurisdictions. This fundamental responsibility implicitly carries an ethical duty to safeguard this sensitive information and to protect the confidentiality of their constituents. This duty has additional significance to the relationships with the health care providers because many health care providers that contribute their data do so voluntarily (ie, few states mandate syndromic surveillance). Moreover, state and local governments have a legitimate interest in ensuring the responsible use of these sensitive data.

These fundamental responsibilities and interests do not end when state and local governments share their data to enable federal agencies to fulfill their legitimate public health interests. Federal partners must keep their state and local partners abreast of their use of syndromic surveillance data and any dissemination of findings. There are important risks in interpreting syndromic surveillance data without understanding local contexts. As prediagnostic EHR data are automatically transmitted in near real time, syndromic surveillance data are messy by design. There can be local variations in submission intervals or health facilities’ data entry conventions that can lead to misleading artifacts in data analyses for the unaware. Additionally, local events—such as a festival drawing out-of-state visitors—could result in expected localized spikes in emergency room or urgent care visits that could be misinterpreted as an emerging public health concern if analysts are unaware of the local context [5]. If federal agencies make decisions from syndromic surveillance data without fully understanding the local context, it is possible that federal actions could interfere with state and local public health activities or create unnecessary communication burdens (ie, states forced to explain or reconcile federal data releases).

State and local health agencies also have important, and sometimes fragile relationships with health care facilities that often voluntarily contribute syndromic surveillance data. Throughout the CSTE study, state and local epidemiologists expressed concern that increasing federal use of granular syndromic surveillance data could endanger these facility relationships if federal communications stopped once the data are received from local agencies without further clarification before any decisions or actions based on these data analyses. Disclosure of a facility’s syndromic data also exposes that facility to certain risks. As 1 key informant said [5]:

we have an understanding with the facilities that contribute data that we’re not just going to release data from a single hospital to the public. So, sometimes it seems like federal users are not as sensitive to that.

Facilities in rural areas are particularly vulnerable to these types of risks as disclosures of granular syndromic surveillance data in rural areas may inadvertently expose the sole rural provider in that area. One informant stated [5]:

I think that the [syndromic] data is just a little bit more fraught than other data, where issues with facility level disclosures,...speaking poorly...about a community without knowing that you are, and that...cultural awareness...that might not be apparent from a federal level...I think that states can bring a lot of value too.

While state and local epidemiologists saw benefits to increased federal access to state and local syndromic surveillance data, access to those data comes with risks that are exacerbated without collaboration and cooperation between federal, state, and local partners [5]. For example, federal interactions with health care providers that bypass state and local partners could threaten local public health relationships with providers that often voluntarily contribute their data to syndromic surveillance programs. If so, these federal actions would also interfere with state and local governments’ fundamental public health mission.

## Intergovernmental Relationships are Critically Important to Data Sharing

Due to the decentralized public health structure inherent to US federalism, strong interagency and intergovernmental relationships are essential for any national public health response. Data sharing between agencies is also critically important in these responses. Indeed, fractures in relationships can be potent data sharing barriers [23]. Relationships are established through time spent communicating and working with each other. Notably, these relationships require trust between partners, and that trust was shaken during the COVID-19 response [5]. One nonfederal key informant noted [5]:

There’s been such a lack of trust that has been reinforced during this response. I think it’s actually going to be harder rather than easier [to permit greater federal access to state or local data]. I say that anyway because I think the NSSP program itself, in its current form, and I think it’s probably important that this gets documented, has been an amazing steward of the data, but the system around it has become less trustworthy and I think the system around it and the system, the way that the response has worked with the states, is now going to impact the program’s ability to do its best work. So, in today’s world, CDC has become less and less willing to really talk to states in pre-decisional ways and help states understand this data is driving this decision, and there’s been a much larger tendency for CDC to make decisions and then just inform states about it in this response. And so, I think pre-COVID, it actually would have been easier rather than harder to implement some of these changes right now, in a way that the states felt good about.

Enabling a truly national public health information system within the context of US federalism requires federal, state, and local partners to forge stronger relationships that will enable greater use of public health data. Done well, collaborating around the use of public health data has the potential to increase communication, common understanding, and teamwork toward a common goal that could increase trust. Importantly, however, public health politization likely creates additional data sharing barriers, as some governments equated the COVID-19 pandemic with successful or unsuccessful governance during a global crisis [24].

## Establish a State and Local Syndromic Surveillance Governance Group

Several state and local epidemiologists suggested openness to creating an NSSP governance group with state and local constituents [5]. Such a governance group should have some decision-making capacities that are currently absent from the community of practice, which functions more as a sounding board or advisory council to the CDC. A governance group with some decision-making authority would create an important check on the expanded federal access to the state and local syndromic surveillance data.

A governance group could have several different benefits for national syndromic surveillance efforts. First, a governance group would be well-positioned to establish a collaborative framework and norms between the federal, state, and local partners. Second, a governance group could establish protocols to help improve transparent communications and collaboration with federal partners regarding ongoing or proposed uses of state and local syndromic data. Third, a governance group could help flag important issues for other state and local partners to monitor, such as proposed federal publications, communications, or policy changes. Finally, a governance group could be empowered to facilitate emergency access to appropriate state and local syndromic surveillance data, greatly reducing the current individual jurisdictional consent burden required in a public health emergency such as the COVID-19 pandemic [5].

The DUAs and other applicable laws should be revised to enable the governance group to function nimbly as public health circumstances necessitate. For example, DUAs could bind state, local, and federal partners to decisions by the governance group to permit expanded emergency access to state and local syndromic surveillance data (and restrict access when emergencies end). This would build flexibility into the DUAs to anticipate and accommodate emergency situations reducing transactional friction while all parties are managing a crisis.

## Develop a Collaborative Framework Between Public Health Surveillance Partners

The US federalism created a decentralized public health system, so policies and agreements establishing a collaborative framework are needed to create a national public health information system, while still recognizing state and local authorities as the primary public health authority. Indeed, the CSTE study identified “improved cross-jurisdiction collaboration efforts” as an important benefit of federal access [5]. This framework would have to carefully establish expectations for federal, state, and local partners and ensure that the legitimate public health interests of all parties are respected.

The CSTE study identified several critical issues that should be addressed in this collaborative framework [5]. Perhaps the most important issue is establishing protocols for communications between syndromic surveillance partners. Currently, communications are not standardized in mode or content. Furthermore, it is not always clear to state and local health departments when the federal government expects responses to their communications (eg, an “FYI” communication vs an investigation inquiry; see Table 1).

**Table 1:** Example of a tiered approach to federal National Syndromic Surveillance System communication response expectations.

Tier	Public health threat	Expected response from states
Tier 3	Low or moderate but only affecting targeted jurisdiction	None
Tier 2	Moderate but interjurisdictional in nature or high but only affecting targeted jurisdiction	Acknowledge receipt
Tier 1	High and interjurisdictional in nature	Response is expected

Given the division of public health responsibilities within the US federalism, these communication ambiguities are additional strains on thin public health resources [5].

A collaborative framework must also (1) establish processes and support for state and local involvement in data analysis and methodological development, (2) provide appropriate acknowledgment, (3) standardize data requests, and (4) restrict federal syndromic surveillance communications with participating health care facilities without the consent of relevant state or local public health partners.

## Establish Appropriate Federal Policy Guardrails for Syndromic Data That Are Shared With Public Health

While many state and local epidemiologists who participated in the CSTE study indicated benefits to greater data sharing with federal partners, many called for strong policy guardrails to ensure appropriate federal uses [5]. To be clear, there are substantial restrictions on syndromic surveillance data currently, but these restrictions prevent legitimate public health data use. If these existing restrictions are loosened to allow legitimate public health uses, then new guardrails need to be established to prevent data misuse and manage risks while still enabling suitable data sharing between agencies. Critically, these sentiments were shared by federal participants in the CSTE study [5]. State and local epidemiologists in the CSTE study indicated support for policies that would (1) establish audit and documentation processes, (2) implement standards and processes to remove access from federal users, and (3) protect sensitive data from public disclosure—such as Freedom of Information Act requests [5]. Additionally, there should be strict limits on sharing NSSP data for nonpublic health purposes. For example, the WHO ethical guidelines strongly advocate against sharing data with agencies that are likely going to take law enforcement action against individuals [19]. Similarly, the facilities that voluntarily contribute their data for the betterment of their communities should not have to fear that their syndromic data will be used against them. The DUAs that address these guardrails would foster stronger relationships of trust in state and local jurisdictions. The existing DUA did not address these issues considering the proposed expanded federal access to state and local data [15].

As data sharing continues to grow between agencies the policies that govern data use need to have well-defined guardrails. In the absence of statutory protections having these guardrails defined in DUAs becomes even more important. This will ensure data are being used in the manner it was intended.

## Significance for Broader Public Health Data Sharing

Data sharing is a persistent challenge for public health agencies [23,25-27]. Although this analysis is limited to the challenges in addressing broader data access to US syndromic surveillance data, it provides insights into data sharing in other contexts. For instance, Aamer Ikram, the executive director of Pakistan’s National Institute of Health recently spoke of the need to support coordinated and integrated public health surveillance, noting that the lessons of the COVID-19 pandemic—good and bad—“must be immediately translated into strategies and policies” [28]. In a 2022 study, The International Association of National Public Health Institutes (IANPHA) identified several jurisdictions where decentralized public health surveillance has complicated the data sharing, coordination, and development of integrated public health data systems [29]. For instance, Mozambique, Canada, and Pakistan all face challenges with local, provincial, and national public health partners that complicate efforts to create integrated surveillance systems [30,31]. The IANPHA report finds that optimal data flow requires systems that permit local and provincial data to flow into compatible national infrastructures supported by formalized data sharing agreements [29]. Accordingly, lessons from this analysis—that deal with facilitating similar data sharing between US local, state, and federal public health partners—could be useful in other international settings.

## Limitations

Our analysis derives from insights from national, state, and local informants based on their experiences in syndromic surveillance practice before and during the pandemic. By the CSTE study’s design, these perspectives skew in favor of the US state and local epidemiologist informants. Moreover, many of the data in the CSTE study are qualitative, which have inherent limitations (eg, representativeness). In our analysis, we sought to consider additional national and systemic considerations, but these additional inputs likely do not eliminate the state or local perspective biases implicit in the CSTE study data used in our analysis. Additionally, there could be additional legal, political, and practical considerations that were not observed within the scope of our analysis.

## Conclusions

The history of US syndromic surveillance is pendular. Every iteration of US syndromic surveillance (ie, Biosense 1.0, BioSense 2.0, and NSSP) was impelled by existing challenges and concerns [7]. However, imperfect responses to those challenges created new issues [7]. For example, to support collaboration between states, BioSense 2.0 shielded syndromic surveillance data from CDC access by using servers operated by the Association of State Health Officials [32,33]. However, this prevented the CDC from assisting with quality assurance and technical assistance [7]. The development of NSSP addressed this issue by having CDC systems once again host syndromic surveillance data but governed by DUAs that established a default of nonsharing between federal, state, and local partners [4,7]. The federal challenges to accessing state and local syndromic surveillance data suggest that the iteration of US syndromic surveillance data will have to wrestle with the policy barriers to intergovernmental public health data sharing.

Broadly, the harsh realities exposed by the COVID-19 pandemic impelled new efforts to improve the national public health data infrastructure. Chief among these efforts is the “Data Modernization Initiative” [34,35]. The CDC’s strategic plan outlines 5 key priorities that are, building the right foundation, accelerating data into action, developing a state-of-the-art workforce, supporting and extending partnerships, and managing change and governance to support new ways of thinking and working [35]. These are important and laudatory goals; however, it is not clear that they will address the challenges that are inherent in the US public health system in the context of federalism.

Future public health challenges such as the COVID-19 pandemic will force the distinct components to once again provide a national response in a decentralized system. State and local governments—having the primary public health responsibility within their jurisdictions—will once again be tasked with the responsibility of collecting data. Federal partners—requiring a national perspective—will once again seek access to these data. Absent a new collaborative framework and data sharing policies, federal agencies seeking access to the needed data will be forced to navigate the governmental bureaucracies of 50 states and hundreds of localities one DUA negotiation at a time. This creates an enormous transactional burden on a public health imperative [19].

Notably, there are substantial efforts to fix these problems. The Coronavirus Aid, Relief, and Economic Security (CARES) Act awarded US $500 million to accelerate data modernization. Part of that funding was intended to upgrade surveillance systems throughout the United States [34,35]. This funding follows the HITECH Act which heavily incentivized public health reporting through the US $35 billion meaningful use program [36]. However, it is here that we must note that these challenges cannot be fixed with new technology or funding alone.

The NSSP is a highly sophisticated public health information system in comparison to other surveillance systems. Nearly all reporting is automated. Reports are transmitted in near real time. Nearly all jurisdictional data are sent to the same data repository. The data sharing challenges are not technical or practical. The data sharing challenges derive solely from relationships between government agencies and policies. These are the challenges that must be addressed to actualize a modern public health information system benefiting the nation as a whole.

## Abbreviations

CARES: Coronavirus Aid, Relief, and Economic Security
CDC: Centers for Disease Control and Prevention
CSTE: Council of State and Territorial Epidemiologists
DUA: data use agreement
EHR: electronic health record
HHS: US Department of Health and Human Services
HITECH: Health Information Technology of Economic and Clinical Health
IANPHA: International Association of National Public Health Institutes
NSSP: National Syndromic Surveillance System
WHO: World Health Organization
